# Identification and Functional Prediction of Poplar Root circRNAs Involved in Treatment With Different Forms of Nitrogen

**DOI:** 10.3389/fpls.2022.941380

**Published:** 2022-07-08

**Authors:** Jing Zhou, Ling-Yu Yang, Chen-Lin Jia, Wen-Guang Shi, Shu-Rong Deng, Zhi-Bin Luo

**Affiliations:** State Key Laboratory of Tree Genetics and Breeding, Key Laboratory of Silviculture of the National Forestry and Grassland Administration, Research Institute of Forestry, Chinese Academy of Forestry, Beijing, China

**Keywords:** circular RNAs, ceRNA regulatory network, *Populus × canescens*, roots, different nitrogen forms

## Abstract

Circular RNAs (circRNAs) are a class of noncoding RNA molecules with ring structures formed by covalent bonds and are commonly present in organisms, playing an important regulatory role in plant growth and development. However, the mechanism of circRNAs in poplar root responses to different forms of nitrogen (N) is still unclear. In this study, high-throughput sequencing was used to identify and predict the function of circRNAs in the roots of poplar exposed to three N forms [1 mM NO_3_^−^ (T1), 0.5 mM NH_4_NO_3_ (T2, control) and 1 mM NH_4_^+^ (T3)]. A total of 2,193 circRNAs were identified, and 37, 24 and 45 differentially expressed circRNAs (DECs) were screened in the T1-T2, T3-T2 and T1-T3 comparisons, respectively. In addition, 30 DECs could act as miRNA sponges, and several of them could bind miRNA family members that play key roles in response to different N forms, indicating their important functions in response to N and plant growth and development. Furthermore, we generated a competing endogenous RNA (ceRNA) regulatory network in poplar roots treated with three N forms. DECs could participate in responses to N in poplar roots through the ceRNA regulatory network, which mainly included N metabolism, amino acid metabolism and synthesis, response to NO_3_^−^ or NH_4_^+^ and remobilization of N. Together, these results provide new insights into the potential role of circRNAs in poplar root responses to different N forms.

## Introduction

Poplars (*Populus* L.) are major fast-growing and high-yield timber tree species and are characterized by rapid growth, strong adaptability, easy reproduction and high productivity; they have important roles in biofuel production, wood processing, the pulp and paper industry and carbon sequestration by forestation (Rennenberg et al., 2010; Castro-Rodríguez et al., 2016; Lu et al., 2019; Zhang et al., 2019). Due to the rapid growth of poplars and their high nutrient consumption, artificial fertilization has become an important management strategy for poplar cultivation (Hu et al., 2020a,b). Nitrogen (N) is an essential mineral that affects the growth, development and yield of poplar plantations (Rennenberg et al., 2010; Luo et al., 2013; Wei et al., 2013). At present, many poplar plantations grow in N-poor areas (Rennenberg et al., 2010). Nitrate (NO_3_^−^) and ammonium (NH_4_^+^), as the main forms of N absorbed from soil by plant roots, have become the most important limiting factors for the growth of woody plants in many regions and seriously restrict the growth and yield of poplar plantations (Kalcsits and Guy, 2016; Zhou et al., 2020). Therefore, in-depth analysis of the molecular physiological mechanisms of how poplar roots absorb, utilize and respond to different N forms is essential to improve N utilization efficiency and reduce fertilizer use.

In recent years, researchers have performed many studies to improve the efficiency of N use in poplars, such as applying exogenous amino acids as N sources (Jiao et al., 2018; Han et al., 2022). Moreover, many N-responsive transcription factors and functional genes have been identified, and their functions in N-associated metabolism and growth have been revealed (Hackenberg et al., 2013; Dash et al., 2015, 2016). With the rapid development of high-throughput sequencing technology and efficient big data technology, an increasing number of nonprotein coding genes, especially microRNAs (miRNAs) and long noncoding RNAs (lncRNAs), have been identified and shown to participate in the N metabolic response of woody plants (Li et al., 2016; Zuluaga et al., 2017; Lu et al., 2019; Fukuda et al., 2020; Zhou and Wu, 2022a). For example, miR167, miR169, miR393 and miR396 were inhibited by N starvation (Gifford et al., 2008; Pant et al., 2009; Vidal et al., 2010). Moreover, 91 differentially expressed lncRNAs (DE-lncRNAs) were identified in poplar xylem under low N treatment and were shown to be involved in wood properties and the physiological processes of poplar (Lu et al., 2019). Our previous study showed that 57 and 36 miRNAs in poplar roots responded to NO_3_^−^ and NH_4_^+^ treatments, respectively (Zhou and Wu, 2022a).

In recent years, circular RNAs (circRNAs), as new endogenous noncoding RNAs, have attracted increased attention from researchers (Ye et al., 2015; Meng et al., 2017; Ng and Pek, 2021). CircRNAs are closed loops, ranging from 10 to 1,000 of bases in length. Compared with linear RNA, circRNAs do not have a 5′-terminal cap structure and 3′-terminal poly-A tail or even a polyadenylated free RNA terminal (Zhao et al., 2019). CircRNAs cannot be degraded by RNases and can be stably preserved (Jeck et al., 2013). These molecules are widely present in all plant tissues and stages of biological development (Ye et al., 2015; Chen et al., 2018; Zhao et al., 2019). CircRNAs can act as miRNA sponges to affect the inhibition of miRNAs on target genes, thus achieving regulation (Hansen et al., 2013; Kulcheski et al., 2016). CircRNA responses to N in plants have also been reported (Liu et al., 2020; Ma et al., 2021). For example, 24 and 22 differentially expressed circRNAs (DECs) were shown to participate in the low N response of roots and leaves of maize (*Zea mays* L.; Ma et al., 2021). In poplar (*Populus* × *canescens*) xylem, 163 DECs were found to participate in the low N response (Liu et al., 2020). However, most studies have focused on N deficit. The differential expression patterns in poplar roots under treatment with different N forms are unclear.

In this study, high-throughput sequencing was used to identify and predict circRNAs in the roots of poplar exposed to three forms of N [1 mM NO_3_^−^ (T1), 0.5 mM NH_4_NO_3_ (T2, control) and 1 mM NH_4_^+^ (T3)] and explored their potential functions in regulating poplar roots. To achieve this purpose, we identified DECs treated with three N forms and annotated the host protein-coding gene functions of these DECs. In addition, the important role of DECs as competing miRNA sponges in regulating the expression of functional genes was analyzed. Moreover, the competing endogenous RNA (ceRNA) regulatory network was constructed, and the functions of differentially expressed target genes (DE-mRNAs) were further annotated. These results provide new insights into the potential role of circRNAs in poplar root responses to different N forms.

## Materials and Methods

### Dataset

The RNA-seq data and small RNA data were derived from our previous study (Zhou and Wu, 2022a). The roots of *P.* × *canescens* (INRA 717-IB4 clone) were used as experimental materials and cultured in Long Ashton (LA) solution (Zhou and Wu, 2022a). After 2 weeks, 9 groups of samples with the same height (6 plants in each group) were selected for treatment with different N forms. Specifically, 500 μM NH_4_NO_3_ in the LA nutrient solution was replaced by 1 mM NaNO_3_ (T1), 500 μM NH_4_NO_3_ (T2, control) and 1 mM NH_4_Cl (T3), and plants were cultured in this treatment solution for 21 days. Each N treatment group contained three biological replicates. All RNA-seq data and small RNA sequences can be downloaded from the Sequence Read Archive (SRA) under project IDs PRJNA631840 and PRJNA631845.

### Data Analysis

Clean reads were obtained using Cutadapt (Martin, 2011) to remove the contaminated adapter and low-quality reads from the raw sequence data. FastQC^1^ was used to verify the sequence quality. Clean reads were mapped to the *P. × canescens* genome sequence^2^ (v1.1) using the Tophat2 (2.0.4) package with default parameters (Kim and Salzberg, 2011). CIRI (Gao et al., 2015) and CIRC Explorer (Zhang et al., 2014, 2016) were used with default parameters to identify circRNAs. The unique circRNAs were first identified by *de novo* assembly. Then, Tophat-Fusion software (Kim and Salzberg, 2011) was used to further compare the *P. × canescens* genome sequence, and reverse spliced reads were identified from the remaining reads (unmapped reads). CircRNAs were considered valid only when recognized by both programs (CIRI and CIRC Explorer software).The R Package-edgeR (Robinson et al., 2010) was used to select differentially expressed circRNAs (DECs) based on the following parameters: |log_2_ (fold change)| values ≥ 1 and values of *p* ≤ 0.05.

### Construction of the ceRNA Regulatory Network and Functional Annotation of DECs

Using PsRobot (Wu et al., 2012), along with the miRNA data obtained in our previous study under three N forms (Zhou and Wu, 2022a), we predicted the interaction between DECs and miRNAs (mismatch score ≤ 2.5, mismatch penalty in the strict matching zone is 1, matching penalty in the nonstrict zone is 0.5 and G: U mismatch penalty is 0.5). Moreover, the miRNA–mRNA interactions were predicted using Target Finder (mismatch score ≤ 2.5). Finally, the circRNA–miRNA–mRNA pairs were screened. Furthermore, circRNAs and mRNAs with significant differences in expression were identified as reliable ceRNA regulatory networks (Liu et al., 2020). Reliable ceRNA regulatory networks were visualized by Cytoscape (V3.6.0).

To determine the potential functional annotation of the DECs, host protein-coding genes of DECs were analyzed by Gene Ontology (GO) functional classification (Conesa et al., 2005), and differentially expressed target genes in the ceRNA regulatory networks were analyzed by GO and Kyoto Encyclopedia of Genes and Genomes (KEGG) analysis (Masoudi-Nejad et al., 2007). Moreover, as described by Thimm et al. (2004), MapMan was used to further analyze the pathways of DE-mRNAs in ceRNA regulatory networks.

### Experimental Validation of circRNAs and Real-Time Quantitative PCR

Genomic DNA of poplar roots was extracted using cetyltrimethylammonium bromide (CTAB). Total RNA was isolated and extracted using a total RNA extraction kit [TRK1001, Lianchuan (LC) Science, Hangzhou, China]. Simultaneously, DNA and ribosomal RNA were removed from total RNA using RNase-free DNase I (E1091, Omega Bio-Tek, Norcross, GA, United States) and ribosomal RNA removal kits (MRZPL116, Illumina, CA, United States), respectively. The RNA was then reverse transcribed using a PrimeScript RT reagent kit (Takara, Dalian, China) according to the manufacturer’s instructions. To confirm that the circRNAs sequenced in poplar roots were actual circRNAs, we used convergent primers (positive control) and divergent primers for PCR (Supplementary Table 1). The PCR products were verified by electrophoresis and sequenced.

To verify the expression of DECs and DE-mRNAs in poplar roots treated with three N forms, RT**–**qPCR was performed on a Light Cycler^R^ 480 Real Time PCR System (Roche, United States) using the SYBR Green PCR kit (TaKaRa) as described by Zhou et al. (2020). The relative expression levels were determined by the 2^–ΔΔCt^ method. The *Actin* gene was used as an endogenous reference gene for circRNAs and targets. All experiments were repeated three times. Specific primer sequences for the amplification of circRNAs and mRNAs are listed in Supplementary Table 1.

## Results

### Identification and Characteristics of circRNAs in Poplar Roots

**Figure 1:**
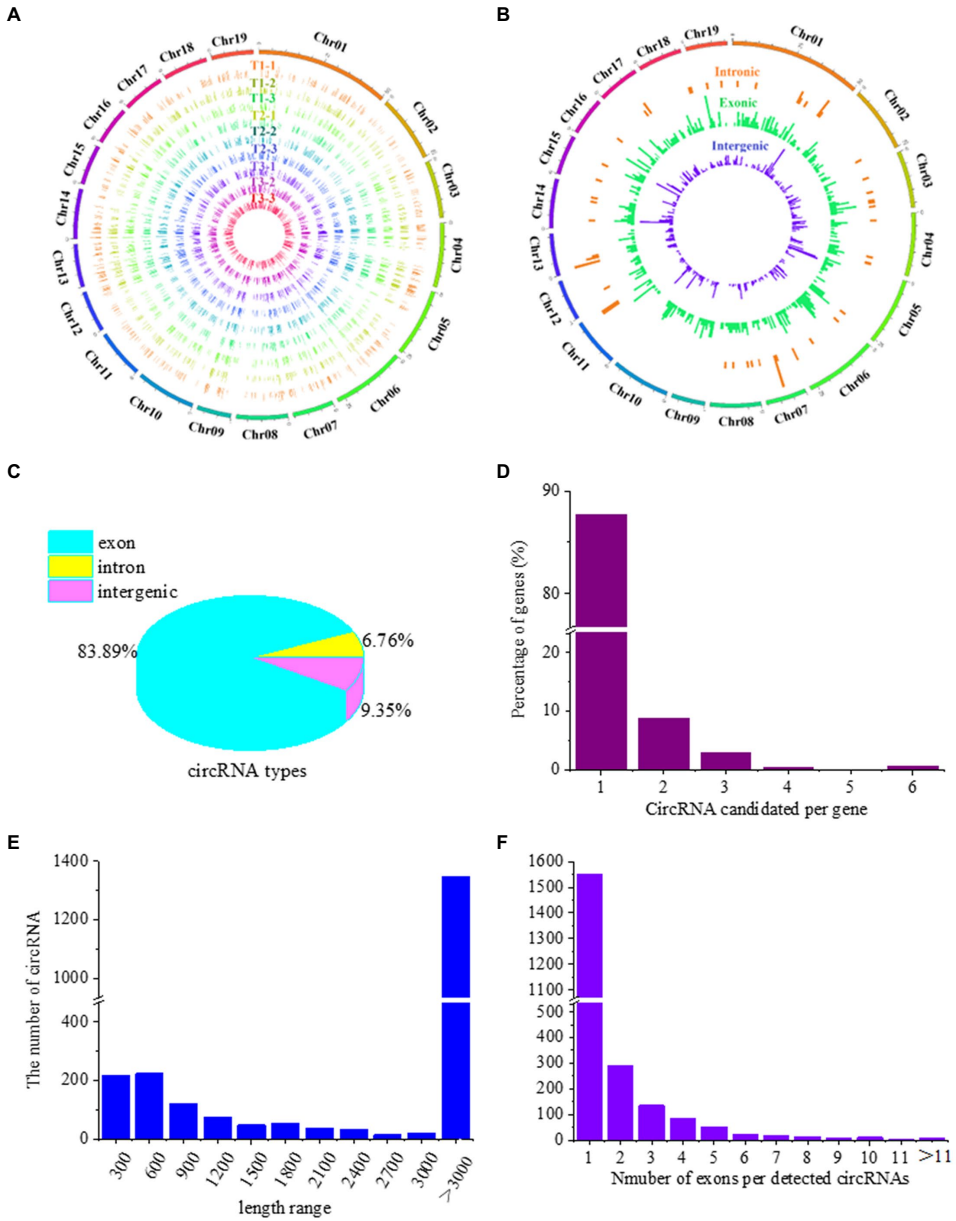
Genomic features of identified circRNAs in the roots of *P. × canescens* treated with NO_3_^−^(T1), NH_4_^+^NO_3_^−^(T2), and NH_4_^+^(T3) conditions for 21 days. **(A)** The expression level of circRNAs along the 19 chromosomes. **(B)** Chromosomal distribution of the three types (exonic, intronic, and intergenic circRNAs). **(C)** The proportion of the three types of identified circRNAs. **(D)** Percentage of hosting protein-coding genes that generated different circRNA candidates. **(E)** Length distribution of circRNAs. **(F)** The number of exons per circRNA.

To determine whether circRNAs are involved in the response of poplar roots to different N forms, we analyzed circRNA expression profiles using sequencing data from nine cDNA libraries constructed from *P. × canescens* roots treated with three N forms in our previous study (Zhou and Wu, 2022a). In total, 17.94, 18.09 and 17.93% of the unmapped reads from the T1, T2 and T3 poplar roots, respectively, were used for circRNA identification (Supplementary Table 2). A total of 2,193 circRNAs were identified, with the number in each sample ranging from 674 to 750 (Supplementary Table 3). The genomic information of circRNAs in the nine libraries was analyzed (Figure 1). A total of 2,193 circRNAs were distributed evenly on 19 chromosomes of poplar (Figure 1A). Similarly, different types of circRNAs were evenly distributed on the 19 chromosomes of poplar (Figure 1B). Among the circRNA types, exonic circRNAs accounted for the largest proportion of read numbers (83.89%). The proportion of intronic circRNAs was the lowest (6.76%), while the proportion of intergenic circRNAs was between that of the other two types (9.35%; Figure 1C). Among the exonic and intronic circRNAs, 762 unique host protein-coding genes were predicted. Among the circRNA and host protein-coding gene pairs, only approximately 87.66% (668) of host protein-coding genes generated one circRNA (Figure 1D). In addition, the length of the circRNAs varied widely, and most circRNA lengths were 100–600 nucleotides and over 3,000 nucleotides (Figure 1E). CircRNAs can have one or more exons, but among the 2,147 potential circRNAs, 1,539 possessed only one exon (Figure 1F).

### Validation of Candidate circRNAs

**Figure 2:**
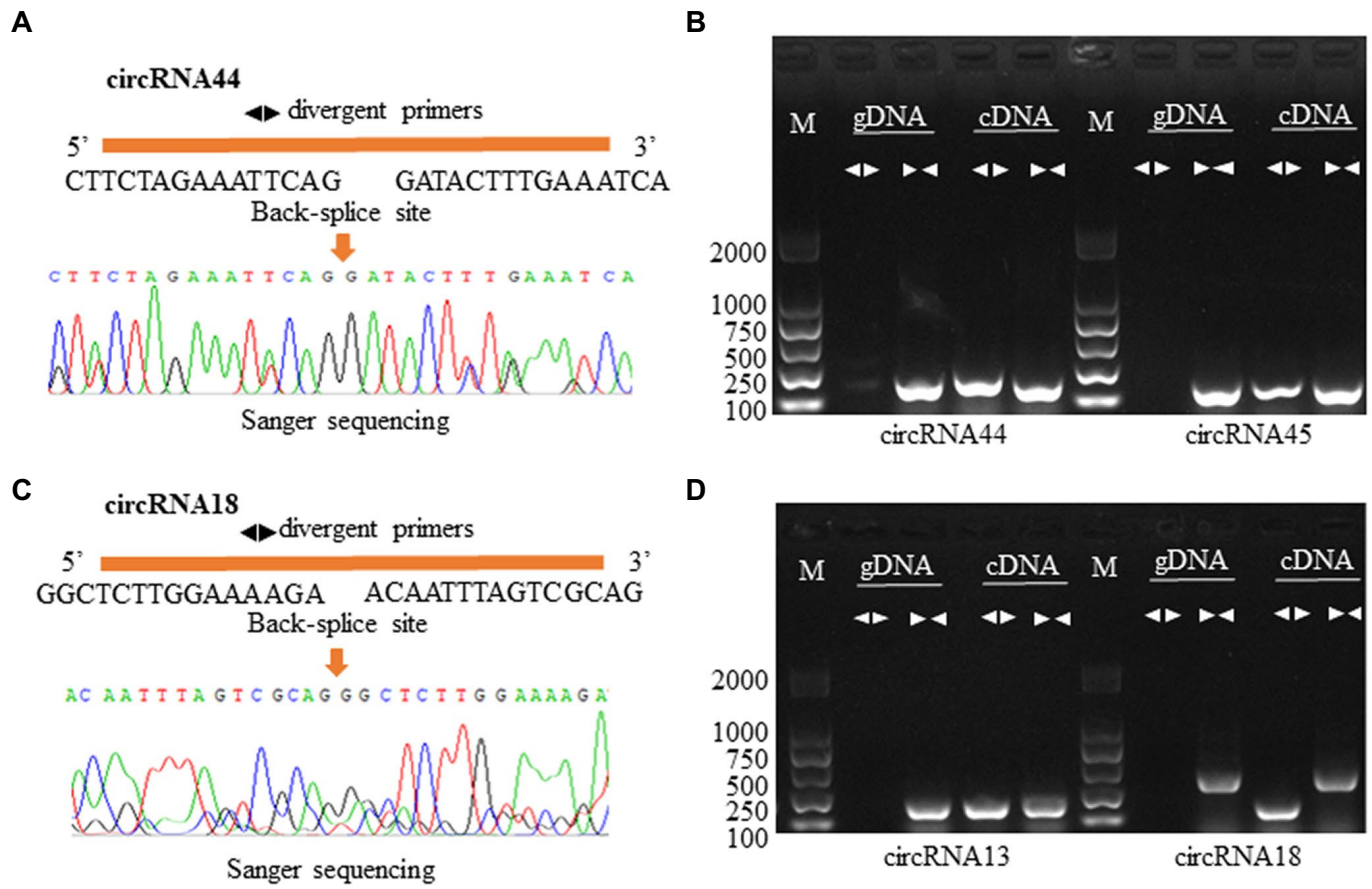
Validation of circRNAs in the roots of P. X canescens. **(A,C)** A detailed depiction of two circRNA circularization and Sanger sequence validation using divergent primers. **(B,D)** PCR amplification results for four predicted circRNAs in cDNA and genomic DNA samples. Convergent and divergent primer sets worked on both cDNA and genomic DNA. M: marker.

To verify the reliability of the circRNA-seq data in this study, we designed divergent and convergent primers for two exonic and intergenic circRNAs to amplify from gDNA and cDNA. As expected, according to the principle of circRNA formation by backsplicing junctions, regardless of whether we examined exonic circRNAs (circRNA44 and circRNA45) or intergenic circular RNA (circRNA18 and circRNA13), divergent primers can only amplify products from cDNAs, while convergent primers amplify products from both cDNA and gDNA (Figures 2B,D). The PCR products were sequenced by Sanger sequencing (Figures 2A,C). This result further proved that the circRNAs obtained by sequencing analysis were reliable.

### DECs Were Identified in Poplar Roots in Response to Different N Forms

**Figure 3:**
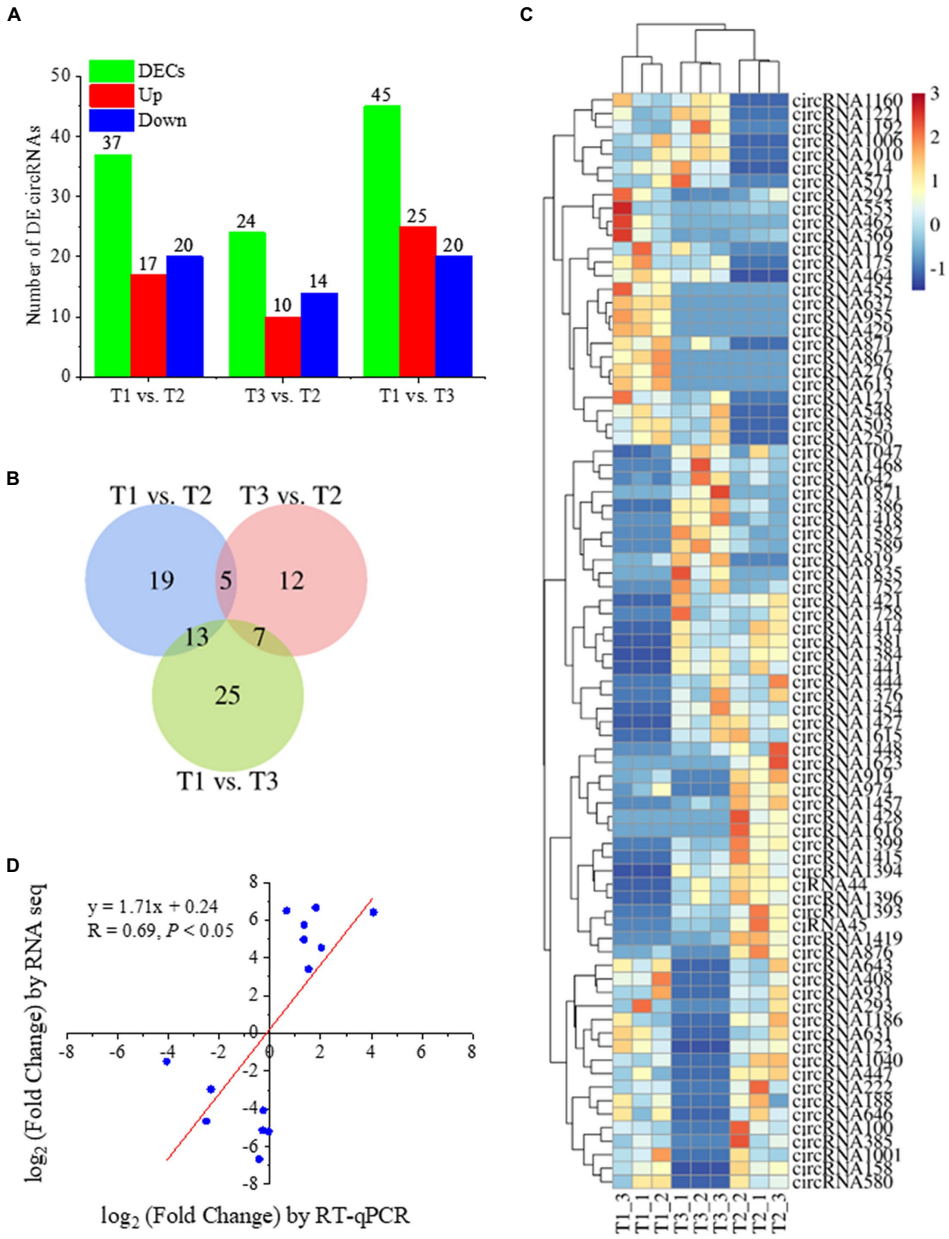
Statistical analysis of identified DECs in Tl, T2, and T3 of *P. × canescens*. **(A)** The number of upregulated and downregulated DECs in each comparison; **(B)** Venn diagram analysis of DECs; **(C)** heatmap of DECs; **(D)** Quantitative RT-qPCR validation of DECs.

To reveal the expression patterns of circRNAs associated with responses to different N forms in poplar roots, we compared the expression of circRNAs upon treatment with three N forms and obtained the DECs in pairwise comparisons (Figure 3; Supplementary Table 4). In the T1 vs. T2 comparison, 37 circRNAs were significantly differentially expressed (*p* value ≤0.05, and |log_2_ (fold change) | ≥ 1). Among them, 17 circRNAs had upregulated expression, and 20 had downregulated expression. We identified 24 DECs in the T3 vs. T2 comparison. Among them, 10 circRNAs showed upregulated expression, and 14 circRNAs exhibited downregulated expression. We also identified 45 DECs in the T1 vs. T3 comparison. Among them, 25 circRNAs showed upregulated expression, and 20 circRNAs exhibited downregulated expression (Figure 3A). Among the DECs identified above, 5 DECs were common in the T1-T2 and T3-T2 comparisons. There were 13 DECs in the T1-T2 and T1-T3 comparisons and 7 DECs in the T3-T2 and T1-T3 comparisons, but no DECs simultaneously existed in the three comparisons (Figure 3B). The heatmap of DECs is shown in Figure 3C. RT**–**qPCR was used to verify the RNA-seq results of 14 randomly selected DECs. The results showed that the expression levels of 14 DECs detected by RT**–**qPCR tended to be consistent with the RNA-seq results (Figure 3D), which indicated that the RNA-seq results were reliable.

To study the function of DECs, we performed GO enrichment analysis of the host protein-coding genes of the DECs (Supplementary Table 5). The results showed that host protein-coding genes of DECs were enriched in biological processes related to amino acid transport (GO:0003333). Moreover, molecular functions associated with amino acid transmembrane transporter activity were enriched (GO:0015171). In addition, biological processes and molecular functions related to amino acid synthesis and catabolic processes, such as lysine catabolic (GO:0019477, GO:0033512) and L-glutamate formation processes (GO:0047131, GO:0047130), were enriched (Supplementary Table 5). These results indicated that host protein-coding genes of DECs were involved in the regulatory process of poplar root response to different N forms.

### DECs May Act as miRNA Sponges in Poplar Root Responses to Different N Forms

CircRNAs have been shown to play a key role in regulating the expression of functional genes as competing miRNA sponges (Hansen et al., 2013). Combined with our previous analysis results of miRNAs treated with three N forms (Zhou and Wu, 2022a), 30 DECs were found to have miRNA binding sites, and 25 corresponding miRNAs were identified (Supplementary Table 6). Among them, circRNA1010 had binding sites for 5 different miRNAs, while 15 different DECs and miRNAs had only one binding site. We also found that several DECs specifically target well-known miRNA family members that play a key role in the N response, such as members of the miR169 and miR396 families (Nova-Franco et al., 2015; Li et al., 2018; Zhou and Wu, 2022a; Supplementary Table 6). Notably, 5 DECs specifically targeting three novel miRNAs (PC-3P-134932_46, PC-3P-2543_1573 and PC-5P-169025_34) were predicted (Supplementary Table 6). The corresponding DEC functions of these miRNAs will be further studied.

### Construction of a ceRNA Regulatory Network in Poplar Roots in Response to Different N Forms

To further explore the function of DECs, we identified 9 DECs that may act as sponges, acting on 11 miRNAs and regulating 77 DE-mRNAs (Supplementary Table 7). The function of each DEC in the regulatory network can be inferred by analyzing the functions of the DE-mRNAs. Therefore, we explored the functions of the DE-mRNAs through GO analysis. The results suggest that the DE-mRNAs were involved in multiple biological processes, such as transcriptional regulation, signal transduction, and response to stress. Most importantly, several DE-mRNAs were also involved in the cysteine biosynthetic process from serine, which affects the synthesis and metabolism of amino acids. Similarly, DE-mRNAs in regulatory networks were associated with multiple cellular component categories, such as nucleoid and integral component of membrane. Molecular function categories were also detected (Supplementary Figure 1). KEGG analysis was performed to further investigate the function of DE-mRNAs (Supplementary Figure 2). The results showed that the DE-mRNAs were enriched in amino acid and sulfur metabolism, such as cysteine and methionine metabolism, cyanoamino acid metabolism, glutathione metabolism and sulfur metabolism. The pentose and glucuronate interconversions pathways, glycosaminoglycan degradation, other glycan degradation and galactose metabolism related to carbon metabolism were also enriched (Supplementary Figure 2). We also used MapMan to classify the DE-mRNAs in the regulatory network into functional categories (Supplementary Table 7). Several functional categories are related to N metabolism, such as amino acid metabolism and synthetic processes.

**Figure 4:**
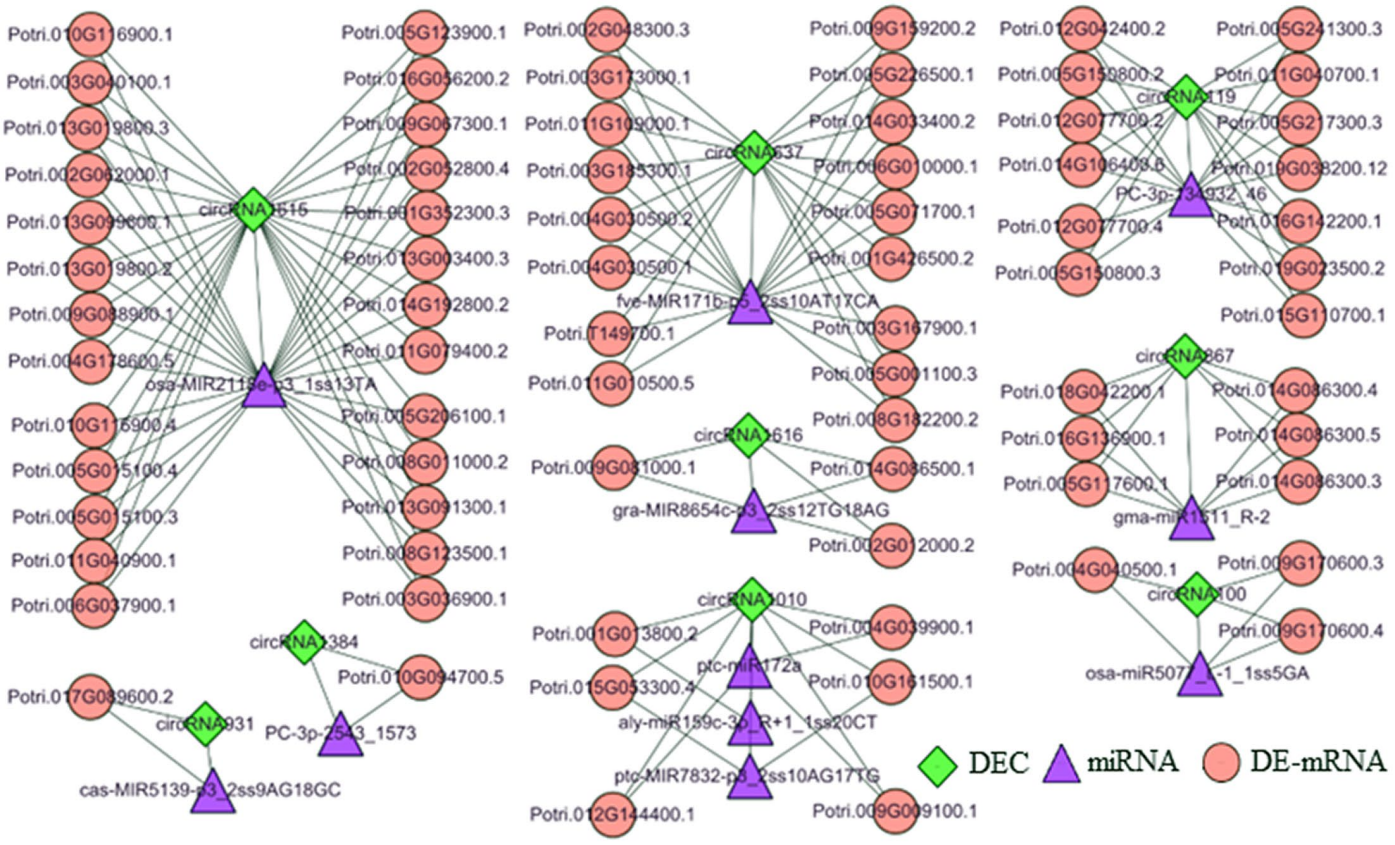
The DEC–miRNA–DEmRNA networks in the roots of *P. × canescens* treated with Tl, T2 and T3. Diamond, triangle, and circular nodes represent circRNAs, miRNAs, and mRNAs, respectively.

To further analyze the function of 9 DECs following treatment with three N forms, we generated a ceRNA regulatory network (Figure 4). In the T1 vs. T2 comparison, circRNA119 expression was upregulated in poplar roots. CircRNA119 may be an endogenous target mimic (eTM) for the novel miRNA PC-3P-134932_46. Upregulation of circRNA119 expression resulted in more binding to PC-3P-134932_46 and less binding to the target gene Potri.019G038200.12 (*S-adenosylmethionine synthetase*, *SAM1*), leading to upregulation of *SAM1* expression levels (Supplementary Table 7). Another circRNA, circRNA867, may be an eTM of gma-miR1511_R-2. Similarly, the upregulation of circRNA867 expression resulted in an increase in gma-miR1511_R-2 binding to circRNA867 and a decrease in its binding to the target genes Potri.014G086300.3, Potri.014G086300.4 and Potri.014G086300.5 (*cysteine synthase D1*, *CYSD1*). Thus, *CYSD1* expression was upregulated (Supplementary Table 7). Another target gene with upregulated expression, Potri.005G117600.1 (*zinc finger family protein*, *GATA15*), was also regulated by circRNA867-gma-miR1511_R-2 pairs. In the T3 vs. T2 comparison, circRNA1010 expression was upregulated in poplar roots. Upregulation of circRNA1010 expression inhibited ptc-miR172a and ptc-MIR7832-p3_2sT10AG17TG targeting Potri.012G144400.1 [*small ubiquitin-like modifier (SUMO) E3 ligase*, *SIZ1*] and Potri.015G053300.4 (*aspartyl protease family protein*, *ASP*), respectively. Another circRNA, circRNA1615, in the T1 vs. T3 comparison had downregulated expression in poplar roots, acting as a sponge to reduce its interaction with osa-MIR2118e-p3, resulting in upregulated expression of 26 target genes (Supplementary Table 7). Clearly, circRNA1615 had a core position in the regulatory network.

**Figure 5:**
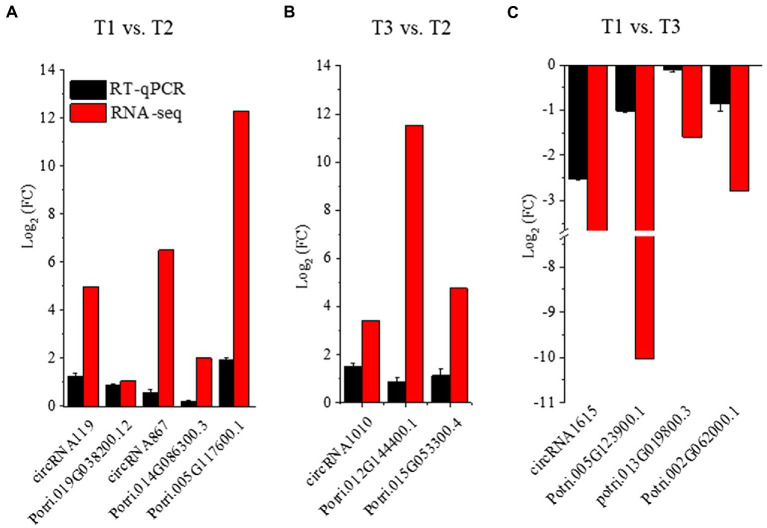
DECs and their possibly regulated mRNAs in the ceRNA network were verified by RT–qPCR in *P. × canescens* root treated with T1, T2 and T3. **(A)**: RT–qPCR results of T1 vs. T2. **(B)**: RT–qPCR results of T3 vs. T2. **(C)**: RT–qPCR results of T1 vs. T3.

To further confirm the expression patterns of DECs and DE-mRNAs in the regulatory network, we used RT**–**qPCR to detect the expression levels of DECs and mRNAs obtained following treatment with three N forms. We found that the expression pattern of the DECs and the corresponding DE-mRNAs tended to be consistent. Moreover, the expression levels of the DECs and mRNAs tended to be consistent with the sequencing results (Figure 5).

## Discussion

Different N forms have a strong influence on the acquisition and utilization of soil N by plant roots (Wei et al., 2013; Jiao et al., 2018; Zhou et al., 2020). Our previous studies have shown that different N forms can change the absorption rate of NO_3_^−^ or NH_4_^+^ from the root tips of woody plants (Zhou et al., 2020; Zhou and Wu, 2022b) and alter the root architecture of poplar trees (Zhou and Wu, 2022a). Moreover, miRNAs, as noncoding RNAs also play a key role in regulating poplar root responses to different N forms (Zhou and Wu, 2022a). CircRNAs, as another noncoding RNA, exist widely in different tissues of plants and play a key role in plant regulation of growth and development and resistance to abiotic stress (Zhang et al., 2020; Huang et al., 2021; Li et al., 2021; Ma et al., 2021). However, circRNAs in poplar root responses to different N forms have not been reported. Therefore, revealing the unique characteristics of circRNAs in poplar roots treated with three N forms is crucial to further understand the response of noncoding regulatory factors to N in woody plants.

### Expression of circRNAs and Functional Analysis of DECs Host Protein-Coding Genes of DECs in Poplar Roots

In this study, circRNAs of poplar roots treated with three N forms were analyzed for the first time. A total of 2,193 potential circRNAs were detected in poplar roots. There were 37 (T1 vs. T2), 24 (T3 vs. T2) and 45 (T1 vs. T3) patients in the three groups treated with the three N forms. However, no DECs simultaneously existed in the three comparisons, indicating that the root response to circRNA treated with different N forms was different. Functional annotation of the host protein-coding genes of the DECs revealed that these genes were associated with amino acid synthesis, decomposition and transport. These results indicated that the DECs were involved in the N response of poplar roots. For example, circRNA44 expression in the roots of *P*. × *canescens* was downregulated after NO_3_^−^ treatment. The transcription of potri.011G167500, a potential host protein-coding gene encoding a*mino acid transmembrane transporter* (*AAP7*), was also downregulated under NO_3_^−^ treatment. *AAP7* is homologous to *Arabidopsis thaliana* AT5G23810, in which this gene is reported to be involved in the uptake of amino acids from xylem (Dolfini et al., 2020).

### DECs Act as miRNA Sponges, and ceRNAs Participate in Poplar Root Responses to Different N Forms

CircRNAs have been reported to act as miRNA sponges, preventing miRNAs from regulating their target genes and regulating gene expression at the posttranscriptional level (Hansen et al., 2013). In this study, we found that several DECs could bind miRNA family members that play key roles in the N response, such as miR169 and miR396 family members. The NO_3_^−^ and N contents in rice overexpressing Osa-miR169o were found to be significantly increased, and the total amino acid content of roots was significantly higher than that of the wild type (Yu et al., 2018). In rice, miR396 inhibits the absorption of NH_4_^+^ by degrading its target gene *growth-regulation factor 4* (*GRF4*), thus inhibiting the growth of rice (Li et al., 2018). Notably, five DECs were specifically predicted for three novel miRNAs (Supplementary Table 6). The corresponding DEC functions of these novel miRNAs will be further studied.

To further study the molecular mechanism of the poplar root response to three N forms, we generated a ceRNA regulatory network. Studies have shown that the DE-mRNAs in the ceRNA regulatory network were involved in the synthesis and metabolism of amino acids in poplar roots, which was consistent with the results in poplar in response to N deficiency (Lu et al., 2019). GO enrichment analysis revealed that the ceRNA networks with circRNAs in poplar wood were related to proline and threonine biosynthetic processes (Liu et al., 2020). Furthermore, DECs were analyzed and may play a key role in the ceRNA regulatory network participating in the response to three N forms. For example, in the T1 vs. T2 comparison, circRNA119 had upregulated expression and combined with more PC-3P-134932_46, resulting in the upregulated expression of *SAM1* in poplar roots. Similarly, the upregulation of circRNA867 expression resulted in an increase in gma-miR1511_R-2 binding to circRNA867, resulting in an increased expression level of *CYSD1s* (Supplementary Table 7). *SAM1* and *CYSD1s* play key roles in the metabolism and synthesis of serine-glycine-cysteine group and are thus used to improve the nutritional quality of crops (Jander and Joshi, 2009). Another target gene with upregulated expression, *GATA15*, was also regulated by the circRNA867-gma-miR1511_R-2 pairs and participated in N metabolism (Bi et al., 2005; Peng et al., 2007). In the T3 vs. T2 comparison, circRNA1010 had upregulated expression and combined with more ptc-MIR7832-p3_2sT10AG17TG, resulting in the upregulated expression of *SIZ1* and *ASP. SIZ1* was involved in growth and development with exogenous NH_4_^+^ supply (Kim et al., 2021). *ASP* can participate in the remobilization of N during leaf senescence (Havé et al., 2017). In the T1 vs. T3 comparison, downregulated circRNA1615 expression stimulated osa-MIR2118e-p3 to 26 target genes, leading to a decreased expression level of 26 target genes in the roots of *P. × canescens* (Supplementary Table 7). Among 26 target genes, Potri.005G123900.1 is highly homologous to *Arabidopsis* AT5G66760, and the target gene encodes *succinate dehydrogenase* (*SDH1-1*), which can improve photosynthesis and promote growth under N-restricted conditions in *A. thaliana* (Fuentes et al., 2011). Potri.013G 019800.3 is highly homologous to *A. thaliana* AT4G24000, and the target gene encodes *CELLULOSE SYNTHASE LIKE G2* (*CSLG2*), which regulates carbon and N metabolism in *A. thaliana* (Bi et al., 2005). Potri.002G062000.1 is highly homologous to *Arabidopsis* AT4G22950. The target gene encoding *MADS-box transcription factor 6* (*MADS6*) regulates lateral root growth and development in *A. thaliana* in response to NO_3_^−^ (Gan et al., 2005). We hypothesize that these DECs, which act as ceRNAs, play a critical role in the response to different N of woody plant roots and plant growth and development.

**Figure 6:**
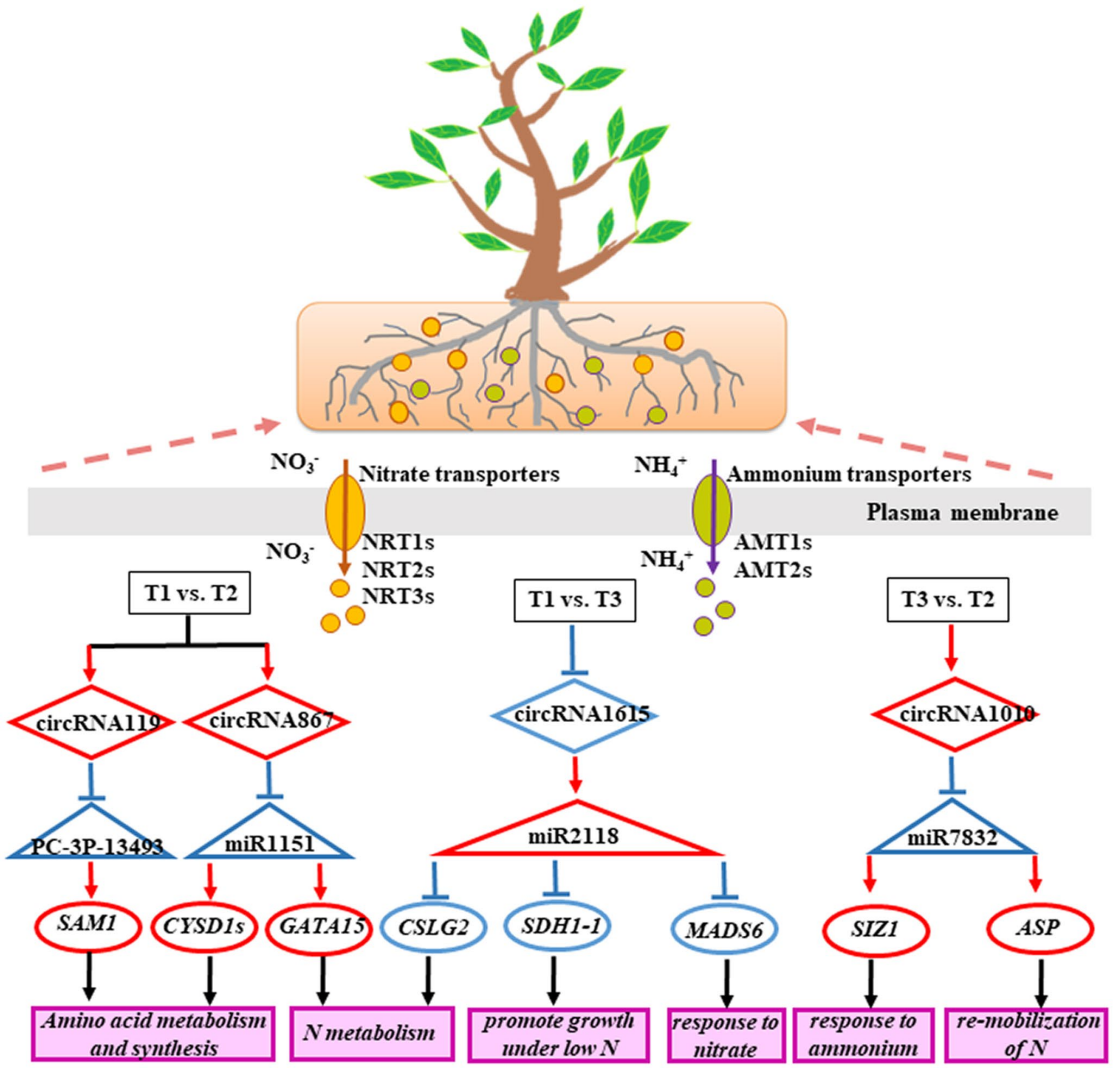
A schematic model of the ceRNA regulatory network involved in the *P. × canescens* root response to different N forms.

In conclusion, our study found that the expression levels of several circRNAs in poplar roots exhibited significant differences under different N forms. Eighty-one DECs were identified in different N form comparisons. Among them, 30 DECs had miRNA binding sites. Moreover, 9 DECs could form a ceRNA regulatory network with 11 miRNAs and 77 target genes. These DECs could participate in the response of poplar roots to N through the ceRNA regulatory network, which mainly included N metabolism, amino acid metabolism and synthesis, response to NO_3_^−^ or NH_4_^+^, and N remobilization (Figure 6). These results provide new ideas for studying circRNAs in response to N in poplar roots to regulate plant growth and development.

## Data Availability Statement

The original contributions presented in the study are included in the article/Supplementary Material, further inquiries can be directed to the corresponding author.

## Author Contributions

JZ conceived the experiment and performed most of the experimental work. JZ, L-YY, and C-LJ performed the experiments and data analyses. JZ, W-GS, S-RD, and Z-BL interpreted the experimental data and wrote the manuscript. All authors contributed to the article and approved the submitted version.

## Funding

This work was supported by the Fundamental Research Funds for the Central Nonprofit Research Institution of CAF (grant no. CAFYBB2022SY007) and the National Natural Science Foundation of China (grant no. 32171739).

## Conflict of Interest

The authors declare that the research was conducted in the absence of any commercial or financial relationships that could be construed as a potential conflict of interest.

## Publisher’s Note

All claims expressed in this article are solely those of the authors and do not necessarily represent those of their affiliated organizations, or those of the publisher, the editors and the reviewers. Any product that may be evaluated in this article, or claim that may be made by its manufacturer, is not guaranteed or endorsed by the publisher.

## Footnotes

^1^ http://www.bioinformatics.babraham.ac.uk/projects/fastqc/

^2^ http://aspendb.uga.edu/index.php/databases/spta-717-genome
