# Supplementary figures and images for: Identification and Functional Prediction of Poplar Root circRNAs Involved in Treatment With Different Forms of Nitrogen
Source: Front Plant Sci. 2022 Jul 8;13:941380. doi: 10.3389/fpls.2022.941380 (PMC9305699; doi:10.3389/fpls.2022.941380)

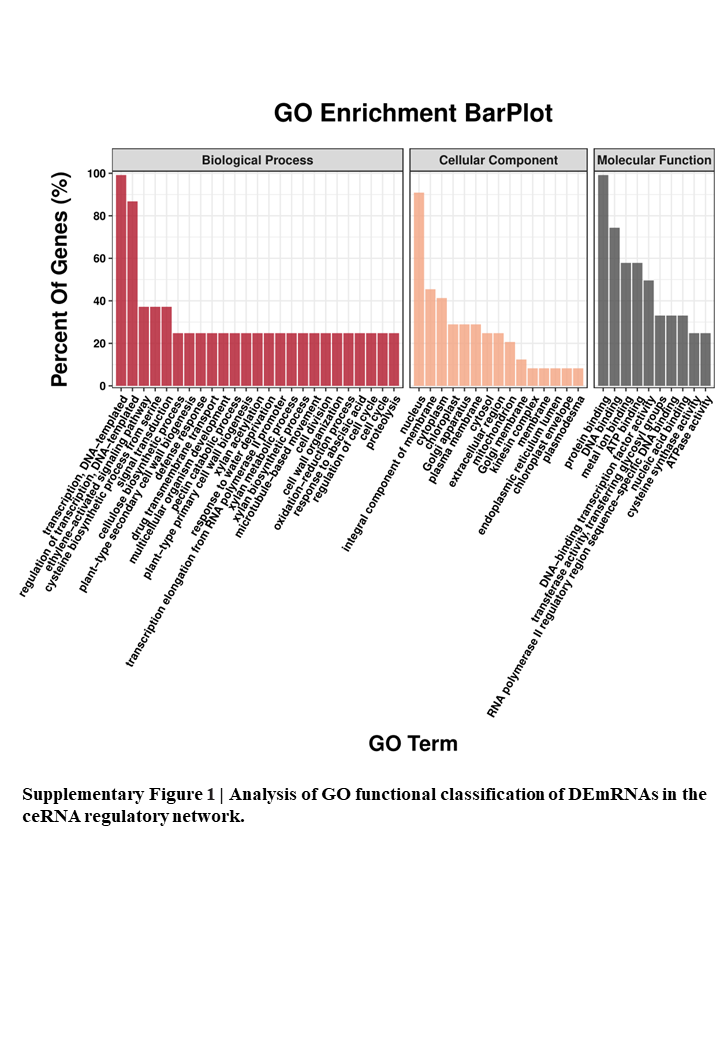

Supplement: Supplementary file 2 [file Image_1.TIF]

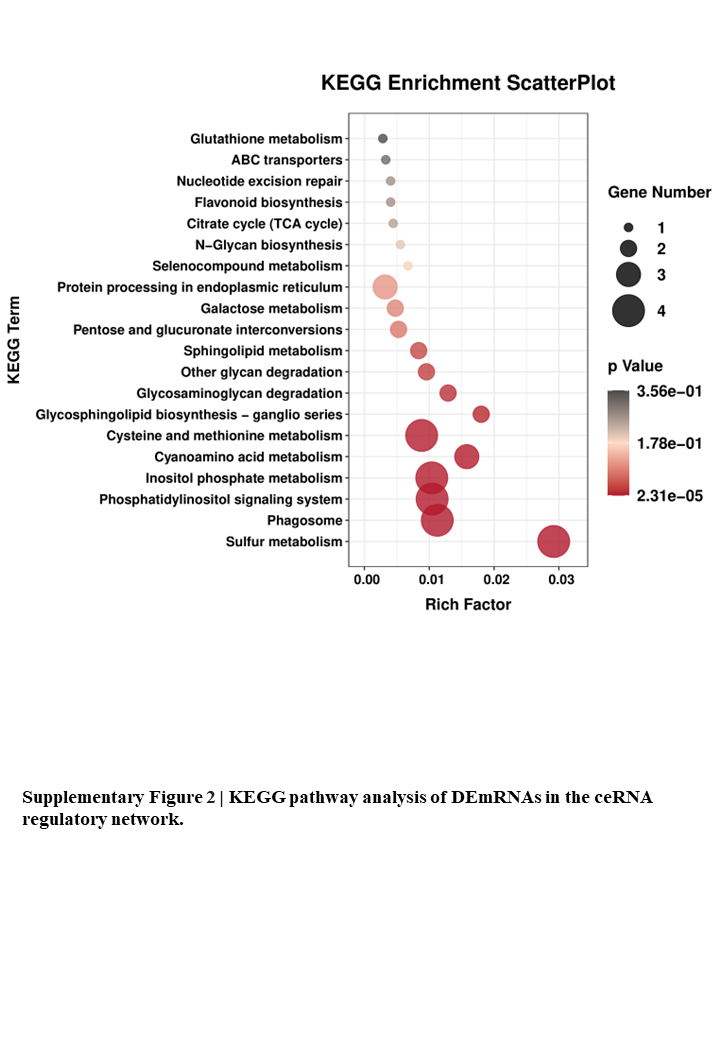

Supplement: Supplementary file 3 [file Image_2.TIF]
